# Insulin resistance: mechanisms and therapeutic interventions

**DOI:** 10.1186/s43556-026-00408-5

**Published:** 2026-02-11

**Authors:** Liuchunyang Yu, Jinxiu Qian, Xiaoyu Li, Meng Tian, Xiuyun Bai, Jue Yang, Rongjun Deng, Cheng Lu, Xiaojuan He, Aiping Lu, Yuanyan Liu

**Affiliations:** 1 School of Chinese Materia Medica, Beijing University of Chinese Medicine, Beijing, China; 2 Institute of Basic Research in Clinical Medicine, China Academy of Chinese Medical Sciences, Beijing, China; 3 School of Chinese Medicine, Hong Kong Baptist University, Kowloon, Hongkong, China

**Keywords:** Insulin resistance, Metabolic dysregulation, Cellular mechanisms, Hepatocellular carcinoma, Therapeutic interventions

## Abstract

Insulin is an important endocrine peptide hormone with pleiotropic effects on metabolic regulation and cellular growth. Insulin resistance (IR), characterized by insensitivity of metabolic tissues to insulin stimulation, has emerged as a major impediment to overall metabolic health. Triggered by multiple environmental factors and genetic predisposition, IR paves the way for several related diseases, including metabolic associated diseases, cardiovascular diseases and cancer. Of note, the liver plays a central role in whole-body metabolism and is the portal encountering high concentrations of insulin. Excess glucose, lipids and the compensatory hyperinsulinemia resulting from IR may collectively impose a huge burden on the liver, driving the progression of chronic liver diseases and fostering a pro-carcinogenic environment by increasing mutagenic susceptibility and angiogenic dysregulation. Better understanding of this mechanistic link is important to highlight the underestimated role of IR in progressive diseases and may contribute to stratified diagnosis and treatment. This review summarizes the risk factors and molecular mechanisms of IR, with a specific focus on its role in carcinogenesis, taking hepatocellular carcinoma (HCC) as an example. Finally, we discuss the effective lifestyle and pharmacological interventions for IR and emphasize the necessity of incorporating IR management into the prevention, stratified diagnosis and treatment of HCC.

## Introduction

Insulin is the master regulator of the integrated anabolic response to nutrient availability, orchestrating efficient energy storage and utilization. However, in modern mechanized societies, lifestyle changes and pervasive imbalances between caloric intake and expenditure have contributed to the rising prevalence of insulin resistance (IR), which is characterized by impaired responsiveness to insulin [1]. IR represents one of the earliest manifestations in a constellation of metabolic disorders, such as obesity, type 2 diabetes (T2DM), and metabolic dysfunction-associated steatotic liver disease (MASLD, previously referred to as non-alcoholic fatty liver disease), leading to a substantial economic and healthcare burden worldwide [2]. Moreover, growing evidence indicates that IR fosters a tumor-favorable microenvironment and is associated with an elevated risk of cancer development [3]. Because IR is a silent pandemic without typical symptoms and is not yet recognized an independent risk factor in the diagnosis of related diseases, it is frequently overlooked in clinical practice [4]. Thus, a comprehensive understanding of IR is pressingly needed.

Insulin performs its functions through binding to the insulin receptor (INSR) and activating downstream signaling pathways [5]. Both genetic and environmental factors play key roles in disrupting a series of signaling transductions in target tissues. Diseases related to IR, such as obesity and T2DM, exhibit a genetic predisposition, and severe IR syndrome has been linked to genetic defects [6]. Additionally, specific lifestyle choices including physical inactivity, circadian misalignment and high-calorie diet can adversely affect metabolic function and overall health, initiating a series of metabolic disturbances that predispose individuals to IR [7]. From cellular and molecular perspectives, chronic energy surplus increases the metabolic burden, which may cause cellular metabolic derangements, including chronic inflammation, oxidative stress, endoplasmic reticulum stress, lipotoxicity, and glucotoxicity. All these can impair insulin responsiveness and blunt critical nodes within the insulin signaling cascades, such as INSR/Insulin receptor substrate (IRS) and phosphoinositide 3-kinase (PI3K)/Akt nodes [8]. IR exhibits distinct manifestations in three key insulin-target tissues—the liver, skeletal muscle, and adipose tissue. Specifically, the liver, as the first organ exposed to high insulin concentrations, is responsible for the majority of insulin clearance and insulin delivery to peripheral tissues, and is subjected to the cumulative metabolic stress exerted by skeletal muscle and adipose tissue [9]. As IR progresses, disordered insulin signaling compromises the handling of glucose and lipids, promoting hepatic lipid accumulation and systemic metabolic abnormalities through interorgan crosstalk [10]. These changes collectively further aggravate hepatic IR, increase metabolic burden, and confer a high risk of hepatic diseases, including hepatocellular carcinoma (HCC) progression [11].

The development of HCC is a multi-step process that mainly occurs upon chronic liver diseases, including viral hepatitis, alcohol-related liver disease and MASLD. Over the years, improved antiviral therapy, accompanied by the rising prevalence of obesity and T2DM, has contributed to a shift in HCC etiology from viral to nonviral causes, particularly those associated with IR and metabolic dysfunction [12, 13]. In the pathological state of IR, persistent metabolic stress and compensatory hyperinsulinemia contribute to the accumulation of genetic mutations, promotion of chromosomal instability and malignant transformation of hepatocytes [14, 15]. These mutations enable cancer cells to retain insulin responsiveness for energy acquisition and cell proliferation [16]. Furthermore, IR continuously influences the angiogenic process, triggering a pro-angiogenic switch in tumors that accounts for the progression of aggressive features and poor prognosis [17].

This review discusses the etiological factors and cellular mechanisms underlying IR, with an emphasis on liver-centered interorgan crosstalk and the contribution of persistent metabolic stress and compensatory hyperinsulinemia to liver carcinogenesis. We further explore IR-related management and therapeutic interventions and highlight the potential of integrating these approaches into the stratified diagnosis, treatment, and prognosis of HCC, which may facilitate the identification of patient subgroups likely to benefit from specific therapies and optimize routine treatment strategies.

## Risk factors and etiological triggers of insulin resistance

**Fig. 1:**
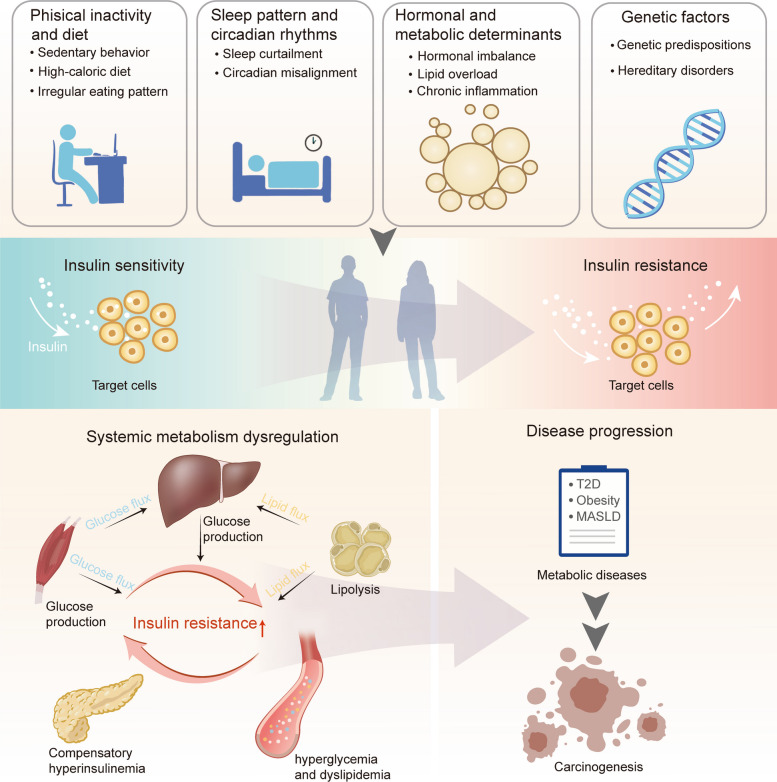
Risk factors and etiological triggers of insulin resistance (IR). Lifestyle choices, hormonal and metabolic influences, and hereditary factors attenuate the biological response to insulin stimulation, leading to unrestrained glucose production and lipolysis in target tissues. To compensate for the disrupted metabolic network induced by IR, insulin secretion from β-cell increases, resulting in hyperinsulinemia which further exacerbates to IR, creating a vicious cycle that promotes the progression of metabolic diseases and carcinogenesis

IR can be defined as the inability of insulin to exert physiological actions, particularly in efficiently regulating glucose storage and utilization in response to nutrient availability. Both nutrient-rich environmental factors and genetic predisposition overwhelm the insulin-mediated adaptive capacity to metabolic challenges, thereby increasing individual susceptibility to IR. This section delineates key etiological determinants of IR development, including lifestyle choices, hormonal and metabolic influences, and hereditary factors. These factors trigger a vicious cycle of mutual reinforcement between IR and disrupted metabolic homeostasis, ultimately driving the progression of IR toward subsequent pathological conditions (Fig. 1).

### Lifestyle factors

Compelling evidence has established that specific lifestyle factors choices, particularly those related to physical inactivity, diet behavior, sleep pattern and circadian rhythms, play a crucial role in the pathogenesis of IR. Mice subjected to 24-h hind-limb cast immobilization to model short-term physical inactivity exhibit accumulation of intramyocellular diacylglycerol and impaired insulin signaling [18]. Another study involving healthy males indicates that 60 days of bed rest with a strictly controlled diet, resulted in an increased fat mass index. While there is no profound metabolic or inflammatory changes occurred, fasting plasma insulin and circulating leptin are markedly elevated, which is a hallmark of metabolic homeostasis disruption and IR onset [19, 20]. Despite meeting physical activity guidelines, an individual who still engage in excessive sedentary behavior may also increase the risk of IR [21]. In sedentary subjects, insulin’s antilipolytic effect in subcutaneous fat is decreased, leading to higher free fatty acids (FFAs) levels [22]. Furthermore, prolonged and interrupted sedentary behavior is increasing recognized as a major component of human movement spectrum that leads to IR, vascular dysfunction, muscle mass and strength loss, and increased adiposity [23].

Diet behaviors, including the overconsumption of high-caloric food and irregular eating patterns, contribute to metabolic dysregulation and the development of IR. For instance, a short-term (7-day) high-calorie, high-fat diet has been shown to induce hyperinsulinemia and blunt skeletal muscle microvascular blood flow, a process critical for glucose disposal in muscle [24]. Another study indicates that 5 days of overconsuming high-caloric diet can trigger hepatic fat accumulation and disrupt brain insulin action [25]. Furthermore, irregular breakfast skipping has been demonstrated to impair vascular endothelial function in the brachial artery [26], and contributed to liver dysfunction [27].

Sleep curtailment has also been linked with IR. For example, a study of 384 adolescents demonstrates that shorter sleep duration and delayed sleep timing are associated with increased homeostatic model assessment of insulin resistance (HOMA-IR), indicating greater IR [28]. More frequent insomnia symptoms are significantly associated with IR and related metabolic traits, such as elevated triglyceride (TG) levels and a higher triglyceride-glucose index [29]. Additionally, the circadian timing system coordinates numerous daily processes, including food intake, energy expenditure, and whole-body insulin sensitivity [30]. Studies show that male mice subjected to an 8 h/8 h light/dark cycle demonstrate impaired insulin sensitivity in the skeletal muscle and liver compared to those on a standard 12 h/12 h cycle [31]. In a prospective study, the rotating night shift workers exhibit impaired glucose tolerance and poor indices of IR, and melatonin treatment does not improve them [32]. Consequently, circadian desynchrony undermines the body’s fundamental ability to adapt to metabolic and environmental changes, leading to disturbances in systemic metabolic homeostasis and insulin sensitivity [33].

### Hormonal and metabolic determinants

Hormonal imbalances and lipid ectopic accumulation can initiate a series of disturbances that predispose individuals to IR. For instance, overnutrition can increase the global sympathetic nervous system activity accompanied with increased plasma norepinephrine, which impair ability of insulin to suppress lipolysis in adipose tissue [34]. Additionally, thyroid hormones are important for muscle formation and fuel energy utilization that improves insulin sensitivity and endurance exercise [35]. In obesity or overweight subjects, the expression of genes related to thyroid hormone action is decreased in skeletal muscle and adipose tissue, suggesting impaired thyroid hormone function [36]. Besides, cortisol and testosterone serve as the major catabolic and anabolic regulators in men, and elevated cortisol together with reduced testosterone induced by sleep loss result in hyperinsulinemia and hyperglycemia [37].

Adipose tissue functions as body’s main lipid reservoir. However, lipid overload due to excessive influx of lipids and glucose leads to both local and systemic inflammation, which in turn diminishes insulin sensitivity [38]. The ectopic deposition of lipids in skeletal muscle, liver and pancreas contributes to tissue-specific IR and pancreatic β-cell dysfunction [39]. For example, lipid accumulation in the liver is closely linked to reduced insulin clearance and impaired insulin function on suppressing hepatic glucose production [40]. Lipid infiltration in skeletal muscle is directly related to declining muscle capacity and is strongly associated with IR [41]. Of note, interorgan communication exacerbates the systemic detrimental effects on insulin signaling with the specific pathways to be discussed in the following Sects. "Cellular and Molecular Mechanisms of Insulin Resistance" and "Tissues- Specific Manifestations and Interorgan Crosstalk of Insulin Resistance".

### Genetic factors

The prevalence of impaired glucose metabolism in offspring is 24.41% with a paternal history of T2DM and 31.13% with a maternal history, and this risk reaches 50.80% when both parents are affected [42]. Offspring of parent with overweight or obesity are linked to higher body mass index (BMI) and adverse metabolic profiles, including elevated fasting insulin levels and HOMA-IR scores [43, 44]. Although the genetic basis underlying these associations remains incompletely understood, advances in genomic approaches have facilitated the identification of common genetic variants associated with IR. Genome-wide association studies have identified several genes involved in impaired insulin signaling and glucose uptake, including *SLC2A4, PPP1R3B, C2D4A, MTNR1B, MTOR, IRS1, PPARG* and *BCL2* [45]. Additionally, studies using the induced pluripotent stem cell derived from IR individuals reveal that *EGR1*, *MFGE8* and the genes enriched in pathways related to cellular oxidative stress are associated with IR [46].

Hereditary disorders are associated with severe IR. For example, myotonic dystrophy exhibits mutations in INSR, resulting endocrine dysfunction and the development of IR, whereas inherited lipodystrophies are strongly associated with IR, hypertriglyceridemia and MASLD [47, 48]. Additionally, mutations in *ALMS1* cause Alström syndrome, which presents with severe metabolic disorders, such as obesity, IR, hyperinsulinemia, hyperleptinemia and hyperlipidemia [49, 50]. Similarly, Werner syndrome, caused by biallelic variants in the *WRN*, is characterized by early-onset diabetes, partial lipodystrophy, severe dyslipidemia and rapidly progressive liver fibrosis [51].

## Cellular and molecular mechanisms of insulin resistance

Diverse steps, from membrane receptors to downstream effectors, are essential to ensure appropriate biological responses to insulin in target tissues. Abnormalities in any of these processes can impair cellular signal transduction, leading to dysregulated insulin signaling. This section will explore the mechanistic links between metabolic disorders and IR by examining defects across the signaling cascade, with a specific focus on the molecular mechanisms through which these disturbances impair critical nodes of insulin signaling.

### The transduction pathway of insulin signaling

**Fig. 2:**
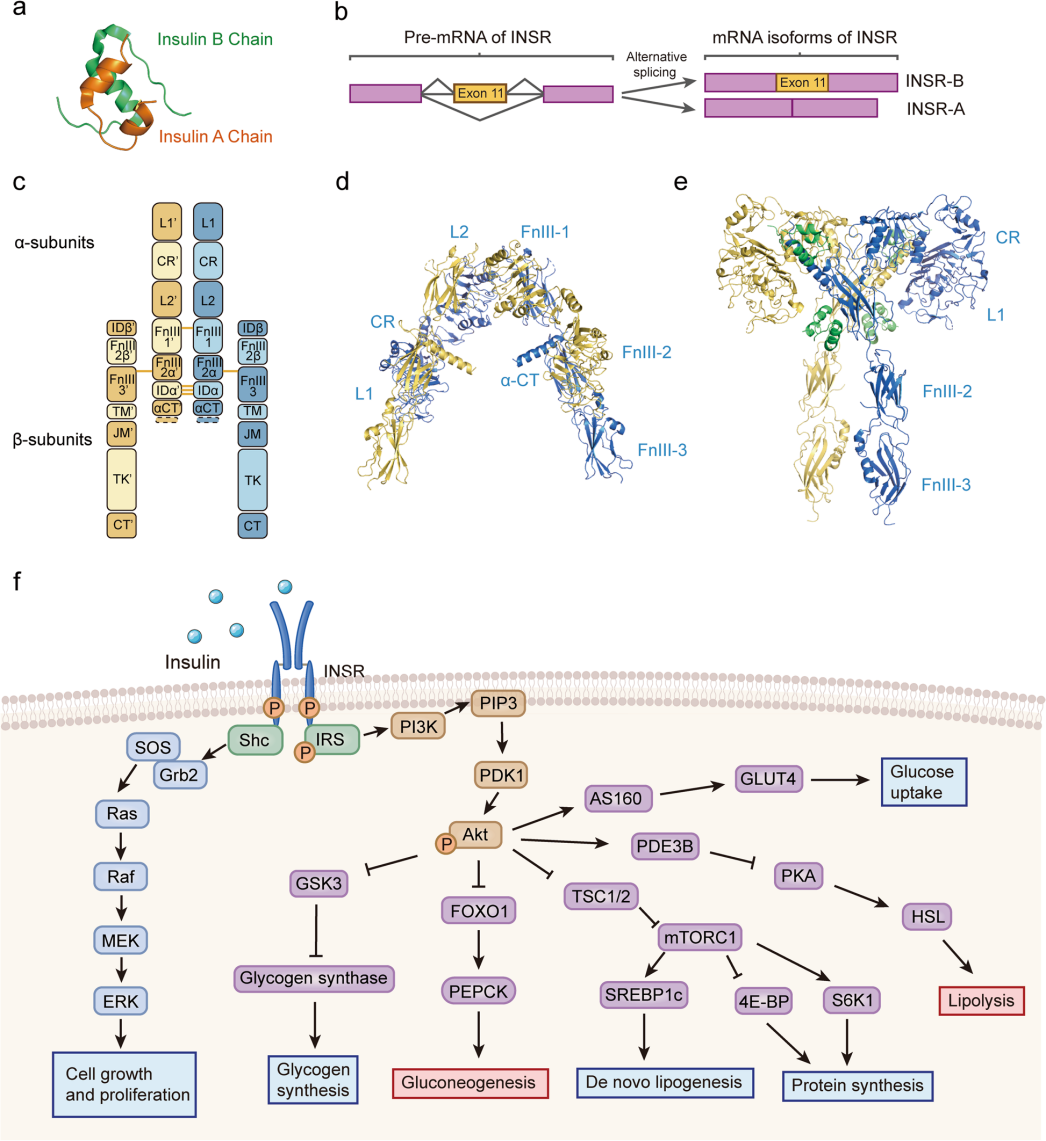
Structure of insulin and insulin receptor (INSR). **a** Structure of insulin (PDB ID: 7ELJ); **b** Alternative splicing of INSR; **c** Protein domains of INSR. INSR-B contains 12-residue at the C-terminus of α-CT; **d** The structure of INSR ectodomain in its Λ-shape (PDB ID: 7SL1); **e** The structure of INSR ectodomain in its T-shape with four insulin molecules (green) (PDB ID: 6PXV). **d** and **e** are based on the short isoform of human INSR; **f** The metabolic and growth-promoting effects of insulin signaling. L1 and L2: leucine-rich repeats; CR: cysteine-rich region; FnIII: fibronectin type III domains; ID: insert domain; CT: C-terminal domain; TM: transmembrane region; JM: juxtamembrane region; TK: tyrosine kinase domain; INSR: insulin receptor; IRS: insulin receptor substrate; Shc: src homology 2 domain-containing transforming protein; SOS: son of sevenless; Grb2: growth factor receptor-bound protein 2; MEK: MAPK/ERK kinase; ERK: extracellular signal-regulated kinase; MAPK: mitogen-activated protein kinase; PI3K: phosphatidylinositol 3-kinase; PIP3: phosphatidylinositol (3,4,5)-trisphosphate; PDK1: 3-phosphoinositide-dependent protein kinase-1; GSK3: glycogen synthase kinase-3; FOXO1: forkhead box protein O1; PEPCK: phosphoenolpyruvate carboxykinase; TSC1/2: tuberous sclerosis protein complex subunit1/2; mTORC1: mechanistic target of rapamycin complex 1; SREBP1c: sterol regulatory element-binding protein-1c; 4E-BP: eukaryotic translation initiation factor 4E-binding protein; S6K1: ribosomal protein S6 kinase beta-1; PDE3B: phosphodiesterase 3B; PKA: protein kinase A; HSL: hormone-sensitive lipase; AS160: Akt substrate of 160 kDa; GLUT4: glucose transporter type 4

Insulin is a peptide hormone composed of two chains linked by disulfide bonds (Fig. 2a). Its physiological functions are mediated by binding to the insulin receptor (INSR), a heterotetramer consisting of two extracellular α-subunits for binding ligands and two transmembrane β-subunits possessing intrinsic tyrosine kinase activity (Fig. 2b, c). Alternative splicing generates two INSR isoforms: INSR-A, which exhibits stronger mitogenic effects and is primarily expressed during prenatal life, and INSR-B, which mainly mediates metabolic effects and predominates in mature insulin-target metabolic tissues [52]. Upon insulin binding, INSR undergoes conformational changes, enabling trans-autophosphorylation of its kinase domains and subsequent phosphorylation of cytosolic substrates including insulin receptor substrate (IRS) and Shc proteins, thus transmitting the signal to multiple intracellular signaling pathways (Fig. 2d-f) [5, 53]. Phosphorylation of IRS allows the recruitment of PI3K, which generates PIP3, leading to the phosphorylation and activation of Akt (also known as protein kinase B). Activated Akt regulates multiple downstream effectors involved in glucose and lipid metabolism, thereby playing a central role in insulin-mediated biological processes [54]. Upon phosphorylation of Shc, it interacts with the adaptor protein Grb2, which forms a complex with the guanine nucleotide exchange factor SOS to active Ras. Subsequently, Ras interacts with the protein kinase Raf, resulting in activation of the mitogen-activated protein kinase (MAPK) cascades, which play an important role in insulin-induced mitogenesis [55, 56].

Among the complex molecules involved in the insulin-signaling pathway, INSR/IRS and PI3K/Akt are the best-defined critical nodes that determine cellular IR. After insulin binding, trans-autophosphorylation of INSR is preferentially initiated at several sites, including Tyr965 and Tyr972 in the juxtamembrane region, and Tyr1146, Tyr1150, Tyr1158 and Tyr1162 in the kinase domain [57]. Conversely, phosphorylation of Thr1160 can dampen the kinase activity of INSR [58]. Insulin-mediated tyrosine phosphorylation of IRS is a key intermediate in insulin signal transduction, whereas serine phosphorylation of IRS acts as a negative feedback control. Various molecular and pathophysiological disturbances, such as inflammation, mitochondrial dysfunction, oxidative stress, ER stress, lipotoxicity, and glucotoxicity, can induce defects in these critical nodes and promote the onset of IR.

### Chronic inflammation and subsequent immune activation

Lipid accumulation is accompanied by enhanced pro-inflammatory cytokine release and activation of inflammatory signaling, thereby contributing to IR. Interleukin-1(IL-1), as a key pro-inflammatory cytokine, regulates inflammatory response through binding to the IL-1 receptor type 1 (IL-1R1). In mice with high-fat diet (HFD) feeding, blocking IL-1 signaling by hepatocyte-specific IL-1R1 knockout increases *IRS-1* and *IRS-2* mRNA and leads to a more pronounced activation of the downstream effector Akt, while retaining insulin sensitivity and glucose tolerance [59]. Additionally, promotion of nuclear factor-κB (NF-κB) activity inhibits the protein expression and phosphorylation of IRS-1 in 3T3-L1 adipocytes [60]. In the liver, NF-κB has been found to inhibit the transcription of phosphodiesterase 3B in response to tumor necrosis factor-α (TNF-α), thereby inhibiting hepatic insulin sensitivity [61]. The inhibitory effects of TNF-α on insulin signaling are also verified. In diabetic hepatocytes model, TNF-α knockdown improves the insulin-stimulated Akt phosphorylation and alleviates palmitate-induced IR [62]. TNF-α treatment in adipocytes induces the increase in p-IRS-1 (Ser307) and decrease in p-IRS-1 (Tyr612), partly by increasing p-JNK and ERK1/2 [63]. Moreover, IL-6 can cause IR by impairing the phosphorylation of INSR and IRS through inducing the expression of suppressor of cytokine signaling 3 (SOCS-3) [64]. SOCS-3 is an insulin-induced negative regulator, which can compete with signal transducer and activator of transcription 5b (STAT5b) at INSR and block IRS-2 tyrosine phosphorylation, resulting in IR [65]. In nutrition-overloaded adipocytes, the excess kynurenine can induce aryl hydrocarbon receptor overexpression, then activating STAT3/IL-6 signaling to reduced p-Akt levels and exacerbates IR [66].

In addition, activated immune cells accumulate within tissues and release more pro-inflammatory signals, reinforcing a vicious cycle of metabolic and immune dysregulation. For example, insulin binding can activate the IRS/PI3K/Akt pathway of macrophages, promoting anti-inflammatory M2 phenotype, but in T2DM, IR leads to increased population of pro-inflammatory M1 macrophages [67]. In HFD mice models, levels of FFA and glucose are elevated and the proportion of M1 macrophages in visceral fat is increased, which are mitigated after tirzepatide treatment [68]. Lipid infiltration in the liver can activates Kupffer cells, leading to the recruitment of monocyte-derived macrophages, which cause inflammation and initiate fibrotic program [69]. Conversely, restoration of anti-inflammatory populations of Kupffer cells demonstrates improved insulin response and hepatic inflammation [70]. Deregulated innate T cells are also involved in IR development. In children with obesity, the frequency of mucosal-associated invariant T cell (MAIT) is increased. The activation of MAIT cells produces elevated IL-17, which impairs insulin-mediated glucose uptake by inhibiting the downstream signaling of insulin, reflected by reduced p-Akt and p-ERK expression [71].

### Mitochondrial dysfunction and oxidative stress

Mitochondria become hyperactive under the nutrition overload and produce more reactive oxygen species (ROS), thereby inducing cellular components damage and triggers transcriptional changes that promote IR. Individuals with T2DM and obesity display a fragmented mitochondrial network in skeletal muscle, accompanied by reduced mitochondrial content and disrupted mitochondrial dynamics, which collectively contribute to diminished oxidative capacity and IR [72]. In mice fed with HFD, the citrate synthase activity, ATP production, mitochondrial respiration and mitochondrial DNA (mtDNA) content in skeletal muscle are significantly decreased [73]. In healthy individuals, skeletal muscle switches from fatty acid oxidation (FAO) to glucose oxidation upon feeding, facilitated by the meal-induced insulin response [74]. Mitochondrial impairment limits this adaptive capacity, leading to an aberrant mobilization and utilization of fat and glucose, thereby increasing FFA concentrations and causing hyperglycemia [75].

Decreased mitochondrial integrity and function are commonly linked to redox imbalances, leading to oxidative stress and impaired insulin signaling [76]. ROS can be produced during fatty acid (FA) β-oxidation in the inner mitochondria membrane as by-products of the electron transport chain or/and generated in glucose metabolism through oxidative phosphorylation, glyceraldehyde autoxidation, and the hexosamine pathway [77]. ROS can activate multiple stress-responsive pathways that interfere with cellular signaling pathways. For example, cytosolic H_2_O_2_ induces activation of IκB kinase (IKK), leading to IκB phosphorylation and sustained NF-κB activation with downstream inflammatory effects [78]. In L6 myotubes, exposure of fructose causes ROS production with diminished translocation of glucose transporter type 4 (GLUT4) and impaired insulin signaling [79]. An appropriate amount of ROS is also required for normal intracellular signaling, including insulin secretion and insulin action. Glucose-mediated physiological ROS production appears to support glucose- stimulated insulin secretion in β-cells, whereas chronic excess ROS results in β-cell dysfunction and impaired insulin release [80]. In skeletal muscle, NADPH oxidase 2 (NOX2)-specific ROS production is necessary for glucose uptake and GLUT4 translocation [81]. ROS can oxidize specific cysteine residues in proteins, thereby influencing their function. For instance, oxidative inactivation of the insulin signaling antagonist protein tyrosine phosphatase 1B (PTP1B) is necessary for effective insulin signaling [82]. Thus, given the fine-tuned regulation of cellular signaling related to insulin function under redox conditions, it is conceivable that excessive ROS production resulting from nutrition overload creates an oxidative stress environment that impairs insulin signaling and contributes to IR.

### Endoplasmic reticulum stress

Endoplasmic reticulum (ER) stress induced by various stimulus can trigger the unfolded protein response (UPR) to activate downstream signaling pathways that are mediated by inositol requiring protein-1α (IRE1α), protein kinase R-like endoplasmic reticulum kinase (PERK), and activating transcription factor-6 (ATF6) [83]. The lipid and carbohydrate metabolic challenges provoke accumulation of misfolded proteins in ER and lead to ER stress. For example, high-saturated fat diet induces hepatic ER stress, accompanied with increased protein expression of ATF6, IRE1 and increased phosphorylation of JNK [84]. The FA treatment also results in ER calcium disequilibrium pool through decreasing sarco/ER Ca^2+^-ATPase protein levels to causes defective calcium transport to the ER [85]. Hyperglycemia has been demonstrated to trigger ER stress-ATF6-CHOP axis and accelerate inflammation [86]. ER stress is associated with impaired insulin signaling. Under FA-induced ER stress, the p-IRS-1 (Tyr896), p-glycogen synthase kinase 3β (GSK3β), and p-Akt are decreased, and the p-IRS-1 (Ser307) is increased, which can be relieved by IRE1α silencing [85, 87]. Methylparaben-induced activation of IRE1α and its downstream effector X-box binding protein-1(XBP1) promotes hepatic inflammation and IR by impairing IRS-1 and Akt activity and decreasing GLUT2 expression [88].

Normally, proteins that fail to reach proper folding are targeted for ubiquitin–proteasome pathway (UPS) or autophagy-lysosome pathway for destruction and degradation. The impairment of these pathways leads to the accumulation of misfolded proteins and ER stress that is related to IR. An abnormal increase in intracellular lipids in the liver may lead to defective autophagy, which further results in IR and ER stress [89]. Furthermore, the proteasome activity is downregulated in the liver of animal models of obesity and T2DM, with the accumulation of ubiquitinated proteins [90]. Proteasome activity is also reduced in adipocytes of obese mice and in 3T3-L1 adipocytes exposed to saturated fatty acid (SFA), which significantly impaired insulin signaling through reducing the expression of IRS-1 and p-Akt [91].

### Lipotoxicity

Accumulation of toxic lipids and intermediates, such as diacylglyceride (DAG) and ceramide, can disrupt cellular homeostasis and cause insulin signaling impairment. The accumulation of DAG in the liver is primarily due to the increased FA uptake and decreased mitochondrial function [92]. The sn-1,2-DAG can directly activate protein kinase C (PKC) ε to translocate to plasma membrane, where the activated PKC ε increases the phosphorylation of Thr1160 on INSR kinase, leading to decreased insulin-dependent activity [93]. Ceramide can potentially increase plasma membrane-sn-1,2-DAG content, which induce hepatic IR through sn-1,2-DAG-PKC ε-INSR phosphorylation pathway [94]. Additionally, ceramide can interfere with insulin signaling cascade by activating protein phosphatase A2 that dephosphorylates Akt, hindering the translocation of Akt to the plasma membrane via activating PKC ζ, and inhibiting mitochondrial respiration [95]. In skeletal muscle, accumulation of C18:0 ceramide can be induced by oxidized phosphatidylcholine and is linked to decreased insulin sensitivity [96]. The C20:0, C22:0 species of ceramide and dihydroceramide also demonstrate inverse relationship to insulin sensitivity [97]. Conversely, exogenous unsaturated ceramide and glucosylceramide can upregulate the IRS/PI3K/Akt pathway to alleviate IR [98].

Excessive consumption of SFA is closely associated with the development of IR. In hepatocytes, palmitate induces miR-183-5p expression, which impairs insulin signaling by downregulating the expression of IRS-1 [99]. Furthermore, under hepatic steatosis conditions, hepatocytes can release small extracellular vesicles enriched in palmitic and stearic acids, which target macrophages and trigger pro-inflammatory responses, inducing hepatic IR [100]. Supplemented with myristic acid can aggravate HFD-induced adipose inflammation, hepatic steatosis and systemic IR in mice [101].

### Glucotoxicity and glycosylation

High carbohydrates intake can induce lipogenesis and steatosis through the activation of multiple lipogenic enzymes [102]. Elevated glucose and lipids contribute to the formation of advanced glycation end products (AGEs), a group of irreversible compounds formed by non-enzymatic glycation and oxidative modification of proteins and lipids [103]. AGEs and their receptor are relevant players in the development of metabolic disorders and IR. AGEs activate ROS mediated ER stress PERK/ forkhead box protein O1 (FOXO1) signaling pathways, inducing the elevated expression of muscle atrophy F-box, skeletal muscle atrophy and IR [104]. Mice fed with high AGEs diet indicate impaired glucose tolerance and hyperinsulinemia, with inhibited carbohydrate catabolism and promoted lipid anabolism, suggesting the key role of AGEs in early IR development [105].

Growing evidence has indicated that abnormal glycosylation participates in the deleterious effects of glucose on IR development. Altered Immunoglobulin G (IgG) N-glycosylation profiles are associated with the severity of IR with increased pro-inflammatory IgG N-glycoforms [106]. Protein tyrosine phosphatase receptor type j (PTPRj) acts as a negative regulator of insulin sensitivity, with its activity potentially regulated by N-glycosylation. This is supported by the finding that HFD-fed mice exhibit increased high-mannose glycosylation on PTPRj and impaired insulin sensitivity [107]. Additionally, increased hexosamine pathway flux has been demonstrated to increase O-GlcNAcylation events, which is linked to inflammatory derangements and the pathogenesis of IR and T2DM [108]. In adipocytes and hepatocytes, the O-GlcNAc transferases recruited by PIP3 at plasma membrane modulate early steps of insulin signaling, such as inhibiting the activity of Akt and IRS-1 [109]. As a putative sensor of systemic metabolism, O-GlcNAcylation fine-tunes insulin signaling kinetics, whereas its aberrant elevation in metabolic organs can compromise the efficiency of insulin signaling [110].

## Tissues-specific manifestations and interorgan crosstalk of insulin resistance

IR exhibits distinct manifestations across main target tissues, which are exacerbated by intertwined interorgan communication, leading to metabolic abnormalities that collectively aggravate systemic IR and may promote the pathogenesis of related diseases. In this section, the manifestation of IR in specific tissues, including pancreatic islets, skeletal muscle, adipose tissue and liver, will be discussed. Of note, given that liver is the first organ exposed to high concentrations of insulin and serves as a major metabolic organ, encumbrance of IR on the liver will be emphasized.

### Pancreatic islets

**Fig. 3:**
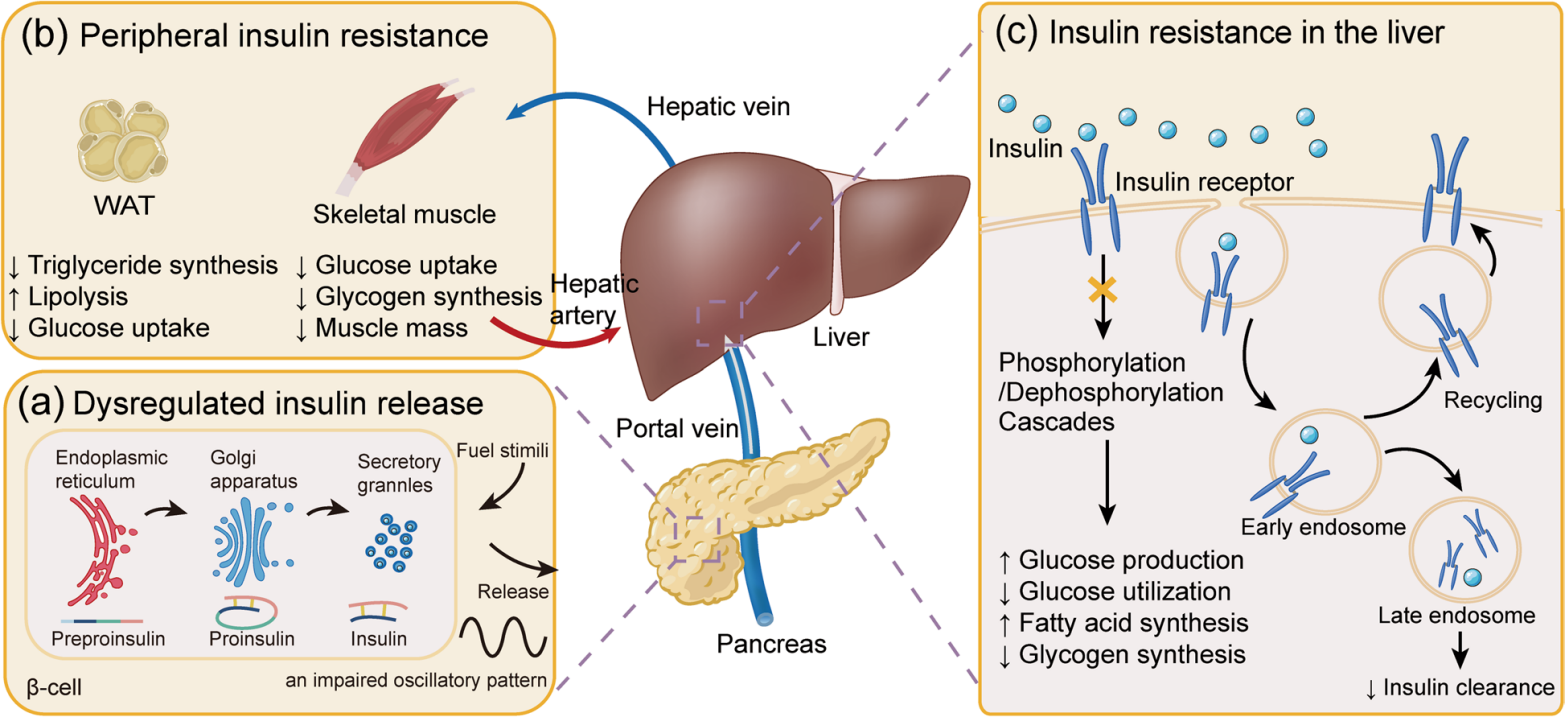
Insulin resistance in different organs. **a** Decreased mature insulin granules and impaired oscillatory insulin release in pancreatic β-cells; **b** Reduced glucose and lipid uptake with unrestrained lipolysis in white adipose tissue (WAT), and decreased glucose uptake and glycogen synthesis in skeletal muscle; **c** Hepatic insulin resistance leads to increased glucose production, persistent lipogenesis, and reduced glycogen synthesis. Declining insulin responsiveness also diminishes insulin clearance

IR conditions damage function of pancreatic islets, mediating β-cell failure and insulin secretion. Pancreas can compensate for IR by increasing β-cell mass and insulin secretion. In obese mice, pancreatic islet hypertrophy, increased α- and β-cell mass and cell apoptosis are observed [111]. However, the decrease in insulin-mediated glucose uptake followed by compensatory hyperinsulinemia ensues to impaired insulin responsiveness, until β-cell activity fails to meet the insulin demand, creating a vicious cycle. Chronic hyperglycemia downregulates the translation of insulin and genes involved in insulin secretory granule formation and exocytosis [112]. The T2DM patients with lipid droplet accumulation in β-cell also demonstrate cell dysfunction and decreased mature insulin granules [113]. Additionally, the altered metabolic conditions and β-cell dysfunction can mask the pulsatility of insulin release and impair the secretory pattern (Fig. 3a). In general, the secretion of insulin is not continuous but in an oscillatory pattern similar to the receptor recycling time, which is thought to avoid downregulation of INSR and preserve the insulin action [114, 115]. In individuals with T2DM, shorter and highly irregular oscillations are observed [116]. Insulin secreted in pulses is more effective at inhibiting glucose production in the liver than continuous insulin secretion [117]. Defects in this oscillatory pattern are likely to blunt hepatocyte responsiveness, which impairs hepatic insulin action and clearance, thereby increasing the amount of insulin that reaches peripheral tissues [118].

### Skeletal muscle

Skeletal muscle is responsible for the major portion of glucose disposal, representing a principal tissue mediating whole body glucose homeostasis. Normally, skeletal muscle accounts for 80% of postprandial glucose uptake, which is then stored as muscle glycogen [119]. In individuals with IR, levels of baseline muscle glycogen are approximately 30% lower, and rate of glycogen synthesis in skeletal muscle is reduced by approximately 50% compared to healthy individual [120]. Lipid exposure to skeletal muscle plays a key role in muscle IR. In obese individuals, increased FA oxidation limits insulin-stimulated glucose utilization, while elevated FA hinder glucose transport into the cells [121]. Lipid accumulation leads to myosteatosis, muscle loss and systemic IR, contributing to sarcopenic obesity, and subsequent progression to full-blown sarcopenia or even cachexia [122]. Notably, ectopic lipid deposition in skeletal muscle has been linked to cancer progression and clinical outcome. For example, study has demonstrated that intramuscular lipid deposition is an independent predictor of mortality in HCC patients [123]. In patients with MASLD and metabolic dysfunction-associated steatohepatitis (MASH, formerly non-alcoholic steatohepatitis), the degree and variability of myosteatosis are independently linked to HCC, irrespectively of liver fibrosis stage [124]. Skeletal muscle abnormalities, such as low skeletal muscle mass and sarcopenia, can affect the outcome and management of HCC, including liver transplantation, resection, ablative, chemoembolization, and systemic therapy [125].

### Adipose tissue

Adipose tissue is classified into white adipose tissue (WAT), where the majority of calories are stored, and brown adipose tissue, which generates heat through thermogenesis [126]. WAT plays a key role in whole-body lipid storage, and insufficient or dysfunctional adipose tissue results in ectopic lipid accumulation in other organs, such as the liver and muscle [127]. IR in WAT is characterized by impaired glucose and FA uptake, reduced TG synthesis and de novo lipogenesis (DNL), and unstrained lipolysis, resulting in elevated plasma FAs (Fig. 3b) [128]. The IR manifestations are also related to the different location of adipose tissues. Visceral adipose tissue (VAT) is more prone to develop IR, due to its heightened hormonal and metabolic activity compared to subcutaneous adipose tissue [129]. Moreover, VAT located in the mesentery and omentum can release substances that drain directly into the liver [130], serving as the main contributor of FFAs in the portal circulation and establishing a close link to hepatic lipid accumulation [131]. Visceral adiposity has been linked to hepatic IR and severity of MASH even in the lean individuals [132]. Conversely, subcutaneous adipose tissue is related to improved insulin sensitivity, and subcutaneous fat transplantation can alleviate glucose intolerance and hepatic TG accumulation [133, 134].

### The interorgan crosstalk collectively exerts stress on the liver

As a central metabolic organ, the liver is particularly vulnerable to disruptions in the main metabolic network caused by systemic IR. Consequently, regardless of whether peripheral IR, hepatic IR itself, or a combination of both, the liver is inevitably subjected to a significant encumbrance. During hepatic IR, there is a defect in insulin-mediated activation of glycogen synthesis and suppression of gluconeogenesis, but an unrestrained increase in hepatic lipogenesis persists (Fig. 3c) [135]. A notable negative correlation is observed between the rate of DNL and insulin sensitivity [136]. This may be explained by the respective abilities of insulin and glucose to upregulate the expression of the key lipogenic transcription factors sterol regulatory element-binding protein 1c (SREBP-1c) and carbohydrate response element-binding protein (ChREBP), which together transcriptionally activate genes involved in DNL [102, 137]. Furthermore, under IR conditions, the upregulation of WD40 repeat-containing protein can facilitate the dephosphorylation of serine/threonine-protein phosphatase 1 to enhance hepatic DNL and promote hepatic steatosis [138].

**Fig. 4:**
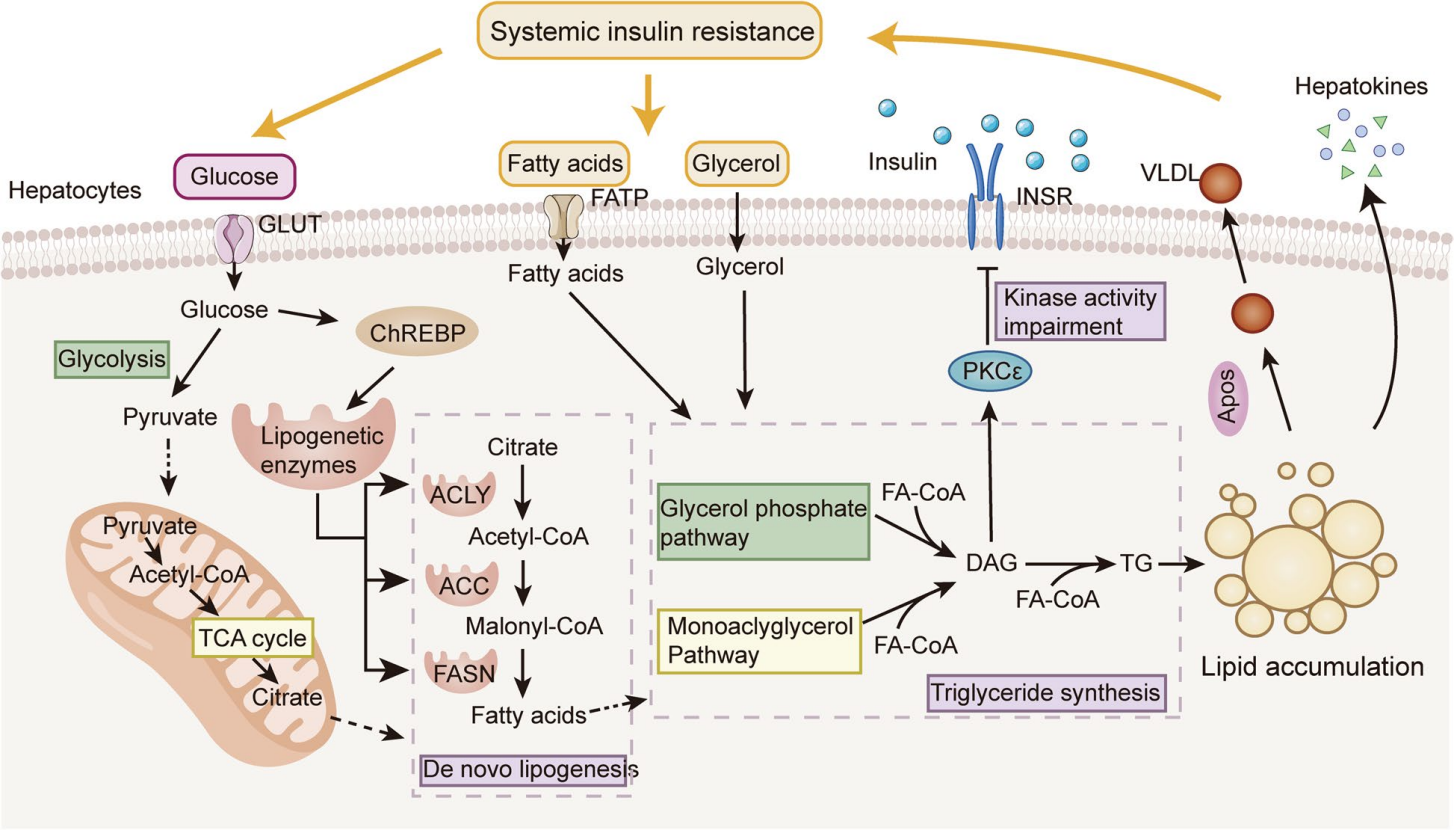
The interaction between liver metabolic disorders and systemic insulin resistance. ACLY: ATP citrate lyase; ACC: acetyl-CoA carboxylase; FASN: fatty acid synthase

The action of insulin in WAT and skeletal muscle is essential for regulating FA turnover in the liver. Muscle IR seems to precede hepatic IR [139], with metabolic dysfunction in the muscle affecting the liver through crosstalk mechanisms. Skeletal muscle IR redirects disposal pattern of ingested carbohydrate, which will be diverted to the liver, leading to enhanced hepatic DNL, followed by hyperlipidemia and a reduction of high-density lipoprotein (HDL) cholesterol concentrations [140]. Moreover, unrestrained lipolysis in adipocytes will increase hepatic TG synthesis and hyperlipidemia due to increased FA esterification [141]. Besides, the increasing FFA and glycerol directed to the liver can enhance the levels of hepatic acetyl CoA and glucose by regulating the activity of pyruvate carboxylase (PC), which can catalyze the conversion of pyruvate to glucose (Fig. 4) [142].

Notably, dysfunctional hepatic metabolism also influences other tissues and disturbs insulin homeostasis. Elevated levels of liver-derived VLDLs contribute to increased circulating TGs and cause peripheral IR by increasing FA availability [143]. After TGs are depleted, lipoprotein particles become enriched with cholesterol, and their accumulation in the plasma membrane will impair GLUT4 translocation and promote IR in skeletal muscle [144]. Additionally, hepatic lipid accumulation can alter hepatokines profile to regulate insulin function in other tissues [145]. For example, fetuin-A is increasingly secreted by the fatty liver, which inhibits β-cells proliferation and disrupts their functional maturation [146]. In decompensated cirrhosis mouse models, liver-secreted fibroblast growth factor 21 (FGF21) mediates liver-muscle crosstalk, inducing sarcopenia [147]. Additionally, as liver is the primary organ of insulin action and clearance, the altered and diseased states can influence its ability to process insulin, which contributes IR. Mice bearing hepatic INSR knockout exhibit glucose intolerance, reduced insulin clearance and pronounced hyperinsulinemia [148]. Furthermore, liver cirrhosis is marked by portal hemodynamic abnormalities, and the reduction in insulin clearance is significantly associated with the degree of the portosystemic shunt [149]. In more malignant state, cancer cachexia in HCC can lead to adipose tissue depletion and alter circulating levels of adipokines, disturbing metabolic and insulin homeostasis [150].

## From insulin resistance to related diseases: taking HCC as a paradigm

IR is implicated in a wide spectrum of diseases, including obesity, T2DM, MASLD, polycystic ovary syndrome and even elevated risks for several types of cancer. The liver’s distinctive vascular architecture, metabolic centrality, and high dependency on insulin for metabolic regulation and growth-promoting underscore its particularly intimate relationship with IR. Therefore, this section examines how IR promotes the transition from chronic liver diseases to HCC, focusing on the carcinogenic role of adipokines and metabolites profiles, and the mechanisms in which hyperinsulinemia and excess nutrient induce genomic instability, oncogenic signaling, and abnormal angiogenesis.

### IR-related microenvironment and carcinogenesis

Adipose tissue represents an active endocrine organ that can release adipokines to communicate with other organs and mediate many biological processes including food intake, energy expenditure, systemic inflammation and insulin sensitivity [151]. Dysregulation of adipokines is linked to IR and has been implicated in hepatic steatosis, inflammation and carcinogenesis [152]. Leptin is the most extensively studied adipokine since its discovery in 1994, acting as an afferent signal that influences the central nervous system to regulate food intake and metabolism through a negative feedback loop [153]. A meta-analysis involving 30 studies show that leptin concentrations are significantly elevated in patients with HCC, and that higher leptin levels are associated with an increased HCC risk [154]. Elevated leptin expression is also related to intratumor microvessel density in patients with HCC [155]. Additionally, it has been suggested that expression of leptin receptor is elevated in liver cancer tissues, which indicates the characteristics of enhancing migration and invasion [156]. Adiponectin is another significant adipokine. HCC patients with elevated levels of adiponectin is linked to shorter overall survival and poorer prognosis [154]. Moreover, in hepatitis B virus (HBV) carriers, elevated adiponectin mediates viral infection and increases the risk of liver cirrhosis, HCC and even liver-related death [157].

In mice models, HFD increases the accumulation of endocannabinoid ligands in adipose tissues, which recruit the specific macrophages that secrete resistin, leading to tissue inflammation and IR [158]. In diabetic patients, elevated resistin levels predict higher risk of HCC [159]. Apelin is an adipokine thought to exert a compensatory role on IR, with circulating levels significantly higher in obese patients with diabetes than in non-obese individuals, similar to the elevation levels of insulin in IR [160]. In addition, apelin contributes to microvessel formation and tumor progression in HCC, and high expression of apelin receptor is associated with worse outcomes [161]. Lipocalin-2 is a circulatory adipokine associated with metabolic disorders such as obesity and T2DM [162]. The biomimetic HCC-on-a-chip analysis indicates that lipocalin-2 plays a key role in remodeling tumor microenvironment that promotes endothelial invasion, drug resistance and immune escape [163].

The elevated flux of FFA and TG observed in IR can impact the lipidome profile, holding potential biological significance in liver carcinogenesis. The proportion of SFA is elevated across the progression of hepatic steatosis and inflammation [164]. SFA exposure, such as palmitic acid, can promote HCC progression through the palmitoylation and stabilization of oncogenic histone deacetylase [165]. Additionally, increased SFA incorporated in the phospholipids of cell membranes can shield cancer cells from oxidative damage, and potentially hinder the uptake of chemotherapeutic drugs [166]. Lipid rafts are sphingolipid- and cholesterol-enriched plasma membrane microdomains that have been implicated as key regulators of signal transduction [167]. In hepatocytes, palmitic acid induces the clustering of lipid rafts, causing cellular injury and dysfunction of insulin signaling [168]. The presence of lipids with saturated chains in lipid rafts allow for more densely packed, ordered lipid arrangements to recruit transmembrane proteins to rafts [169]. In this way, lipid rafts acting as centralized platforms can enhance the binding of membrane-anchored receptors to their ligands and promote the downstream cascades that are essential for survival, death and metastasis of cancer cells [170].

### Oncogenic mechanisms of IR

#### Genomic instability and malignant transformation

ROS and reactive aldehydes generated under the conditions of elevated glucose and lipid levels have been reported to directly promote DNA damage and inhibit DNA repair. Highly reactive hydroxyl radicals attack DNA by adding to the double bonds of DNA bases or by abstracting hydrogen atoms from the methyl group of thymine and from each C-H bonds of 2-deoxyribose [171]. Guanine, the most readily oxidized of the four DNA bases, is converted to 8-oxo-7,8-dihydroguanine, which matches with adenine and thereby causes G:C-T:A transversions, leading to mutagenesis and epigenetic alterations [172].

Polyunsaturated FAs are very vulnerable to oxidative attack by ROS, leading to lipid peroxidation [173]. The main products of lipid peroxidation include malondialdehyde (MDA), acrolein, crotonaldehyde and 4-hydroxynonenal (4-HNE) [174], which can promote mutagenic effects and disrupt the biological activity of insulin. MDA can react with DNA to form adducts with deoxyguanosine and deoxyadenosine. The MDA-deoxyguanosine adduct induces the sequence alterations through transversions and transitions of base pair substitutions [175]. Acrolein can react with DNA to form mutagenic adducts with deoxyguanosine, which further enhance oxidative DNA damage-induced mutagenesis [176]. Acrolein also inhibits nucleotide and base excision repair pathway, and mismatches repair pathway by causing the degradation of DNA repair proteins [176]. Crotonaldehyde reacts with deoxyguanosine in DNA to form adducts, which can cause interstrand crosslinks or DNA–protein crosslinks within duplex [177]. The 4-HNE-DNA adducts are preferentially formed with the third base of codon 249 in *TP53* gene, suggesting potential impacts on tumor initiation and promotion [178]. Besides, 4-HNE can form adducts with insulin, significantly disrupting its biological activity and contributing to the pathogenesis of IR [179].

#### Abnormal activation of insulin proliferative signaling

In the setting of IR, the mitogenic effects of insulin appear to be undisturbed and may even be upregulated due to hyperinsulinemia and genetic mutations involved in insulin signaling and INSR. Increased insulin concentrations upregulate the activation of farnesyltransferase (FTase), leading to higher levels of farnesylated Ras, which enhance the mitogenic responsiveness of cells [15]. Insulin’s influence on cellular growth has been observed in various cell types [180, 181], as it stimulates DNA synthesis and promotes cell cycle progression [182]. Studies have shown that insulin treatments lead to elevated levels of cyclin D1 and c-Myc [183]. Another study demonstrates that insulin simulation significantly reduces the expression of the *Ccng2*, which encodes cyclin G2, by more than 30-fold [184]. Cyclin G2 promotes cell cycle arrest in tumor cells, and its reduced expression is linked to more aggressive phenotype and poorer prognosis [185]. Dysregulated cyclin level or aberrant cyclin-dependent kinase activation directly contributes to cancer hallmarks by enabling uncontrolled proliferation [186]. Thus, continuous insulin stimulation may allow cells to override the restriction of cell-cycle checkpoints, leading to aberrant mitosis.

**Fig. 5:**
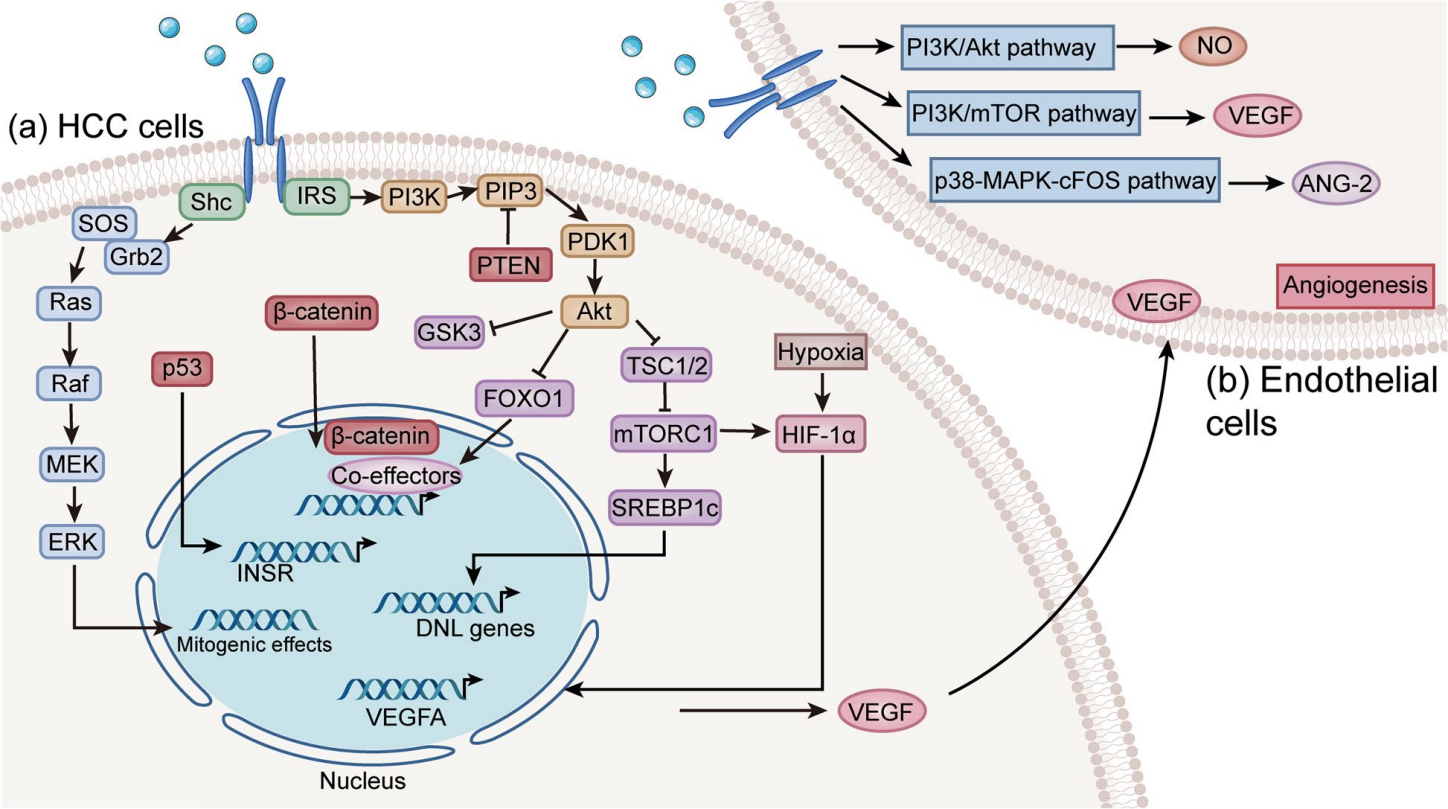
**a** Insulin signaling and genetic mutations in HCC cells. The mutated p53 strongly stimulates INSR promoter and increases the expression of INSR. Activation of Wnt-β-catenin signaling can regulate the phosphorylated level of IRS and insulin sensitivity. In the nucleus, β-catenin interacts with various co-effectors to regulate diverse cellular processes. **b** The potential role of insulin signaling in endothelial cells proliferation and angiogenesis. In endothelial cells, insulin can increase the production of NO through a PI3K/Akt-dependent pathway and elevate the expression of pro-angiogenic factors, including VEGF, ANG-1, and ANG-2. VEGF: vascular endothelial growth factor; ANG: angiopoietin; HCC: hepatocellular carcinoma; INSR: insulin receptor; IRS: insulin receptor substrate; Shc: src homology 2 domain-containing transforming protein; SOS: son of sevenless; Grb2: growth factor receptor-bound protein 2; MEK: MAPK/ERK kinase; ERK: extracellular signal-regulated kinase; MAPK: mitogen-activated protein kinase; NO: nitric oxide; HIF-1α: hypoxia inducible factor-1α; PI3K: phosphatidylinositol 3-kinase; PIP3: phosphatidylinositol (3,4,5)-trisphosphate; PDK1: 3-phosphoinositide-dependent protein kinase-1; TSC1/2: tuberous sclerosis protein complex subunit1/2; mTORC1: mechanistic target of rapamycin complex 1; SREBP1c: sterol regulatory element-binding protein-1c; FOXO1: forkhead box protein O1; DNL: de novo lipogenesis; GSK3: glycogen synthase kinase-3; PTEN: phosphatase and tensin homolog

Genetic alterations observed in HCC affect genes encoding several proteins within the insulin signaling pathway. Aberrant activation of these pathways and loss of negative regulators shift insulin signaling from normal systemic endocrine regulation to a pro-tumorigenic process (Fig. 5). *TP53* and *CTNNB1* are the prevalent mutations in HCC, affecting 25% to 30% of HCC patients [187]. In cancer cell lines with missense *TP53* mutations, insulin promotes cell proliferation and enhances invasion capabilities, which is diminished when endogenous mutant p53 is depleted [188]. Additionally, mutated p53 strongly stimulates INSR promoter and regulates the expression of INSR [189]. Gain-of-function mutations in *CTNNB1* and loss-of-function mutations in *AXIN1* can result in deregulated Wnt-β-catenin signaling, which drives the expression of a number of genes, thereby creating a network of molecules that promotes tumorigenesis [190]. In hepatocytes, Wnt-β-catenin signaling activation is reported to affect phosphorylated levels of IRS, causing IR and promoting lipogenesis [191]. Under oxidative stress conditions, β-catenin directly interacts with FOXO1, transcriptionally enhancing its target genes [192]. These genes are vital for mediating insulin’s metabolic effects and are implicated in regulating the metabolic reprogramming that tumor cells undergo to sustain growth [193, 194].

As outlined above, insulin binding to its receptor triggers the activation of the PI3K and Ras/MAPK signaling cascades. Chronic exposure to elevated insulin levels in the liver leads to further activation of PI3K signaling, which can drive malignant transformation [195]. In normal tissue, the PI3K/Akt pathway is negatively regulated by phosphatase and tensin homologue (PTEN) [196]. However, reduced PTEN expression is frequently observed in HCC, which is related to larger tumor size and shorter overall survival in patients [197]. Moreover, the loss of PTEN disrupts multiple facets of lipid metabolism in the liver. In mice with liver-specific PTEN deficiency, significant increases in TG and cholesteryl ester levels are observed [198]. The Ras/MAPK cascades catalyze the phosphorylation of cytoplasmic substrates or nuclear transcription factors, mediating mitogenic effects and committing the cell to either a proliferative or differentiation cycle [199]. Persistent activation of effector molecules in the MAPK signaling plays a significant role in the pathogenesis of HCC [200].

The interplay between insulin and insulin-like growth factor (IGF) signaling is critical in cancer cell proliferation. Insulin and IGFs can share their receptors and several overlapping downstream signaling pathways, due to the high homology of both ligands and receptors [201]. In HCC, this pathway is frequently dysregulated. For instance, IGF-2 is often overexpressed, which is significantly associated with hepatic progenitor cell features and an aggressive phenotype [202]. Meanwhile, a retrospective cohort study indicates that INSR overexpression is frequently observed in HCC patients and correlates with worse pathological parameters and survival [203]. Moreover, the abnormal alternative splicing of INSR in HCC leads to an elevated ratio of INSR-A/INSR-B, which is associated with enhanced cancer stemness and tumor progression [204]. Notably, INSR-A exhibits higher binding affinity for IGF-2 [205]. Frequent INSR overexpression with a predominance of INSR-A isoform in HCC synergistically increases cellular responsiveness to the mitogenic and proliferative signaling of insulin and IGF-2, thereby fueling cancer progression [206].

#### Angiogenesis and metastasis

The liver’s unique vascular pattern is one of the fundamental features of its organization. Because nutrients and oxygen delivered by blood vessels support hepatic function, angiogenesis is essential for liver regeneration. However, dysregulated angiogenesis can allow tumors to thrive, contributing to malignant diseases [207]. Pathological angiogenesis is a hallmark of HCC, and subtypes characterized by angiogenic activation and vascular invasion typically exhibit histological features of aggressiveness and poor prognosis [208]. Tumor neovascularization requires the recruitment and proliferation of endothelial cells to form the inner lining of blood vessels within the tumor stroma. Endothelial cells also express INSR and respond to the metabolic and growth actions of insulin [209]. At pathophysiological concentrations, insulin stimulates the migration and tube formation of endothelial cells, indicating that insulin may function as an angiogenic promoter [210]. Insulin has also been shown to induce the proliferation of human umbilical vein endothelial cells (HUVECs) [211]. Overexpression of INSR in HUVECs leads to an approximately threefold increase in nitric oxide (NO) production in response to insulin [212]. Endogenous NO can promote arteriolar dilation to maintain tumor blood flow, thereby promoting tumor progression and invasion [213]. NO also stimulates angiogenesis and mediates the effects of angiogenic factors. It has been found that NO production plays a significant role in the growth-promoting effect of vascular endothelial growth factor (VEGF) and appears to be a crucial downstream component of VEGF signaling [214]. Besides, NO exhibits mutagenic properties, and sustained exposure to high NO concentrations promotes carcinogenesis through NO-mediated DNA damage and impairment of DNA repair [215].

Angiogenic factors tightly regulate angiogenesis, inducing the transition from dormant, avascular hyperplasia to an expanding vascularized tumor [216]. HCC cells and pericytes can secret angiogenic factors, such as VEGF and angiopoietin (ANG), to promote the development of new vessels [217]. VEGF is crucial for the growth and migration of HCC cells and significantly contributes to hepatocarcinogenesis [218]. Its expression progressively increases from low-grade dysplastic nodules to high-grade dysplastic nodules and contributes to the transition to early HCC [219]. In sinusoidal endothelial cells, insulin significantly increases the expression of VEGF and its receptors in regenerating rat liver following partial hepatectomy [220]. Moreover, the upstream signaling pathways of VEGF are regulated by insulin. Hypoxic conditions in tumor can increase intracellular hypoxia-inducible factor (HIF) -1α, which induces VEGF expression [221]. Insulin has been shown to induce the formation of the HIF-1α/aryl hydrocarbon receptor nuclear translocator (ARNT) transcription complex in HCC cell lines [222]. This insulin-mediated induction of HIF-1α, shared with the effects of hypoxia, depends on the PI3K/mTOR pathway, thereby leading to increased VEGF expression [223]. ANG also has critical roles in angiogenesis and exhibits potential clinical and prognostic significance in HCC [224]. Insulin has been demonstrated to promote vascular wound healing partially by regulating ANG-1, which enhances pericyte coverage and supports the reconstruction of the vascular basement membrane, indicating that insulin is a potent driver of vascular maturation [225]. Under hyperinsulinemia conditions, insulin also upregulates ANG-2 mRNA and protein expression by mediating p38-MAPK-cFOS pathway in endothelial cells, thereby promoting endothelial inflammation to initiate angiogenesis [226].

### Controversies and unresolved issues in IR-HCC link

Although IR is well established as a key driver of the progression from chronic liver disease to HCC [227], there is no universal consensus on whether targeting IR confers therapeutic benefit in patients with HCC. For example, adding metformin to routine sorafenib demonstrates poor treatment response in HCC patients with high expression of VEGF and HIF-1α [228]. In advanced HCC patients receiving first-line immunotherapy, metformin use has been associated with a trend toward worse clinical outcomes, although this did not reach statistical significance [229]. A probable explanation is that, during tumor progression, the accumulation of genetic mutations and disruption of signaling pathways prevent cancer cells from relying on the initial IR-related environment for proliferation, limiting the therapeutic effect of interventions targeting IR. Consequently, responses to such strategies in HCC patients are difficult to predict, given the considerable heterogeneity in tumor etiology, disease stage, health states, and prior treatments. However, interventions aimed at mitigating IR have shown promise in reducing the burden of chronic liver diseases and slowing their progression to HCC. This highlights the importance of IR management as a potential direction for prevention of HCC and emphasizes stratified therapeutic approaches in patients with manifest HCC.

## Therapeutic interventions and management strategies

Although it is challenging to defeat IR entirely, lifestyle modifications and pharmacological approaches have been used as effective strategies to improve insulin sensitivity and glycemic control. Early and sustained intervention can delay disease progression and reduce long-term complications, with potential benefits in impeding liver disease progression and reducing the risk of HCC. This section summarizes established and emerging strategies for IR treatment and broaden the management of IR as a potential component in counteracting malignant diseases.

### Lifestyle interventions

Lifestyle interventions, such as dietary modifications, physical activity, and weight management can improve insulin sensitivity and alleviate liver diseases. Mice with 20% calorie restriction demonstrate enhanced peripheral insulin sensitivity, lower insulin requirement to maintain euglycemia, and prolonged pancreatic β-cell longevity [230]. Medical nutritional therapy plays a crucial role in improving hepatic steatosis and fibrosis [231]. In humans, energy restriction and exercise intervention for 10 months significantly reduce body weight, fat mass and liver injury in MASH individuals, and improve peripheral insulin sensitivity [232]. Regular physical activity also predicts lower risk of HCC [233]. Moreover, adhering to healthy diet principles like those of the Mediterranean diet and a low-fat diet is beneficial for reducing liver enzyme levels and intrahepatic fat [234]. Likewise, Mediterranean diet and dietary patterns representing anti-inflammatory potential are associated with reduced risk of both fatty liver and HCC [235], while inflammatory and insulinemic diet are associated with increased HCC risk [236]. Dietary intake of unsaturated FA is associated with reduced risk of developing IR [237]. Overall, pursuit of a healthy lifestyle is crucial for maintaining proper insulin sensitivity and liver function, which is important for reducing the risk of HCC development.

### Pharmacological approaches

While no medications are specifically approved to treat IR alone, several anti-diabetic medications can lower plasma glucose, improve metabolic parameters, and enhance insulin sensitivity. Notably, some of these drugs have shown promise in ameliorating MASLD/MASH and reducing HCC risk (Table 1). Metformin has served as the first-line medication for T2DM. It lowers glucose levels through targeting liver and gut and shows anti-inflammatory properties [242]. Several studies have highlighted its potential in preventing HCC development. A nationwide case–control study has demonstrated that metformin use can decrease the risk of HCC in a dose-dependent manner, with each additional year of metformin treatment leading to a 7% reduction in HCC risk among diabetic patients [265]. Besides, after successful antiviral therapy in patients with diabetes and chronic hepatitis C virus (HCV) infection, metformin treatment greatly reduces HCC incidence [238]. Metformin can mediate the expression of PD-L1 and directly rescue tumor-infiltrating CD8 T lymphocytes, thereby enhancing cancer immunotherapy [266, 267].

**Table 1:** The application of anti-diabetic drugs in HCC prevention and treatment

Drug type	Mechanism	Drug name	In pre-HCC hepatic diseases	In HCC	Reference
Biguanides	The representative drug metformin can lower glucose levels by reducing hepatic gluconeogenesis, inhibit the intestinal absorption of dietary glucose and regulate brown adipocyte metabolism	Metformin	It alleviates hepatic steatosis in HFD mouse models by regulating lipophagy and necroptosis	It reduces HCC incidence in patients with diabetes and HCV after successful antiviral therapy	[238–242]
			It shows no benefit in preventing hepatic decompensation in patients with diabetes and compensated cirrhosis	It is associated with reduced all-cause mortality (HR: 0.589, 95% CI: 0.454–0.763) and lower risk of tumor progression (HR: 0.667, 95% CI: 0.526–0.845) in patients with T2DM undergoing TACE	
TZDs	TZDs activate the PPARγ and improve the response to insulin	Pioglitazone	It alleviates liver histopathology, liver enzymes and reduces blood lipids in both non-diabetic and diabetic MASLD patients	Use of pioglitazone is associated with lower liver cancer incidence (OR: 0.83, 95% CI: 0.72–0.95) in T2DM patients	[243, 244]
		Rosiglitazone	A 1-year treatment of rosiglitazone reduces hepatic steatosis and improves insulin sensitivity, but with no additional histological benefit beyond 1 year of treatment	Use of rosiglitazone is associated with lower liver cancer incidence (OR: 0.73, 95% CI: 0.65–0.81) in T2DM patients	[243, 245]
DPP4i	DPP4i Inhibits the DPP4 enzyme, which increases the body’s natural GLP-1 and GIP levels	Sitagliptin	Sitagliptin treatment improves glucose metabolism but does not reduce the intrahepatic lipid content in MASLD patients with T2DM	Sitagliptin as neoadjuvant therapy in HCC patients exhibits potential in improving antitumor immunity	[246–248]
			Sitagliptin treatment with lifestyle modification reduces fibrosis scores and AST levels in MASLD subjects		
		Linagliptin	A 52-week linagliptin treatment in MASLD patients with T2DM shows increase in serum leptin levels and reduction in hepatic steatosis and but not superior to metformin	Linagliptin suppresses cell proliferation in Hep3B cell lines and inhibits tumor growth in xenograft mice models with NRF2 inhibitor	[249, 250]
		Alogliptin	Alogliptin can alleviate chemically induced liver fibrosis in rat models by suppressing inflammation, oxidative stress and fibrogenesis	Alogliptin administration improves survival rate and reduces levels of tumor markers in DEN-induced HCC rat models	[251, 252]
GLP-1 RA	Mimic GLP-1 and directly activate its receptor	Liraglutide	Liraglutide improves hepatic steatosis, glycemic markers and lipid profiles but not liver stiffness in MASLD patients	Liraglutide treatment ameliorates hepatic steatosis and inflammation and suppresses carcinogenesis in STZ- and HFD-induced diabetes and MASH mice models	[253, 254]
		Semaglutide	Semaglutide is associated with a lower risk of major adverse liver outcomes (aHR, 0.73; 95% CI 0.60–0.88), which is a composite end point consisting of decompensated cirrhosis, HCC, and liver transplantation in patients with MASLD and T2DM		[255]
		Tirzepatide	Tirzepatide treatment improves MASH without worsening of fibrosis in individuals with MASH and moderate or severe fibrosis	Tirzepatide ameliorates MASH, hepatic fibrosis and tumorigenesis in STZ + HFD mice	[256, 257]
SGLT2i	SGLT2i inhibits SGLT2 to prevent renal glucose reabsorption from the glomerular filtrate, thereby lowering the plasma glucose level and promoting excretion of glucose in the urine	Canagliflozin	Canagliflozin treatment improves liver biochemistry and beneficially affects liver fibrosis in T2DM patients	Canagliflozin reduces the expression of β-catenin in HCC cells, delays tumor growth and improves the survival of HCC bearing mice	[258–261]
			Canagliflozin reduces hepatic lipid accumulation in MASLD and ALD mouse models		
		Empagliflozin	Empagliflozin treatment is associated with improved liver steatosis and fibrosis and decreased body weight and abdominal fat in patients with MASLD and T2DM	Combination of empagliflozin with metformin shows apoptotic and anti-inflammatory potential in HCC mice models	[262–264]
			Combination of pioglitazone with empagliflozin reduces liver fat and stiffness and prevents MASLD in T2DM individuals		

*TZDs* thiazolidinediones, *DPP4i* dipeptidyl peptidase 4 inhibitor, *GLP-1 RA* glucagon-like peptide 1 receptor agonist, *GIP* glucose-dependent insulinotropic polypeptide, *SGLT2i* sodium-glucose co-transporter 2 inhibitor, *HFD* high fat diet, *HCV* hepatitis C virus, *PPARγ* peroxisome proliferator-activated receptor γ, *aHR* adjusted hazard ratio, *CI* confidence interval, *OR* odds ratio, *MASLD* metabolic dysfunction-associated steatotic liver disease, *MASH* metabolic-Associated Steatohepatitis, *ALD* alcoholic liver disease, *T2DM* type 2 diabetes, *TACE* transarterial chemoembolization, *HCC* hepatocellular carcinoma, *STZ* streptozotocin, *DEN* diethylnitrosamine, *AST* aspartate transaminase

Thiazolidinediones (TZDs) are potent insulin sensitizers found to be ligands for PPARγ, which exhibits a variety of effects the regulation of glucose and lipid homeostasis as well as modulating inflammation [268, 269]. A meta-analysis has reported that treatment with rosiglitazone or pioglitazone significantly reduced incidence of liver cancer [243]. Rosiglitazone treatment also enhances the chemosensitivity to Adriamycin in insulin-resistant HCC cell line models [270].

Dipeptidyl peptidase 4 inhibitor (DPP4i) has been available for treating T2DM since 2006 [271]. It inhibits the catalytic activity of DPP4 to reduce glucagon-like peptide 1 (GLP-1) degradation, thus enhancing the anti-hyperglycemic effects of GLP-1 and improving glycemic control [271]. In the animal models of HCC, DPP4i has been reported to activate NK cell and T-cell chemotaxis, thereby suppressing tumor growth and inhibiting tumor angiogenesis [272]. DPP4i treatment has demonstrated a significantly reduced HCC risk in patients with T2DM and HCV infection [273]. In animal models, DPP4i effectively prevents the progression from steatosis to inflammation and tumor development [274]. However, another study indicates that DPP4i users are associated with higher risks of decompensated cirrhosis and hepatic failure, suggesting this anti-diabetic approach may not suitable for patients with cirrhosis [275]. Using GLP-1 receptor agonist (GLP-1 RA) is a more direct pathway to activate the GLP-1 receptor signaling, which is efficacious for T2DM and weight loss [276, 277]. In patients with MASLD and diabetes, GLP-1RA treatment is associated with a reduced risk of progression to cirrhosis and related complications, but it does not lower rates of hepatic decompensation or HCC among patients who already have cirrhosis, underscoring the importance of initiating treatment earlier in the disease course [278].

The sodium-glucose co-transporter 2 (SGLT2) is primarily expressed in the proximal tubule, and accounts for 80%-90% of glucose reabsorption. Inhibiting SGLT2 decreases the glucose reabsorption capacity of the proximal tubule and improves glycemic control over time [279]. SGLT2 inhibitors can improve IR and decrease visceral fat mass in patients with diabetes [280]. SGLT2 inhibitors also reduce the risk of kidney failure and cardiovascular diseases [281]. In HCC treatment, SGLT2 inhibitor canagliflozin demonstrates the ability to suppress the proliferation of HCC cell lines and delay the tumor growth in xenografted tumor model [258].

Enzyme α-glucosidase in small intestine is responsible for the hydrolyses of dietary carbohydrates. The α-glucosidase inhibitor (AGI) can improve hyperglycemia by reducing carbohydrate digestion [282]. In patients with T2DM and liver cirrhosis, AGI treatment is associated with a lower risk of all-cause mortality and HCC [283]. In contrast, in insulin-treated diabetic patients, the use of AGIs has been associated with a higher mortality risk [284]. Sulfonylureas are effective and affordable anti-diabetic drugs that stimulate insulin secretion by targeting the sulfonylurea receptor 1 on the β-cells, but their use is curtailed by secondary effects and risk of hypoglycemia [285, 286]. In patients with T2DM and MASLD, glimepiride use can improve hepatic steatosis and ballooning but it is not superior to SGLT2i tofogliflozin [287]. In contrast, studies have found that diabetic patients using glyburide, face a sevenfold higher risk of developing HCC compared to non-users [288]. Another report shows a 1.7-fold increased risk of HCC development in patients with T2DM, and among the different anti-diabetic medications, glimepiride use is associated with a significantly elevated HCC risk, whereas gliclazide use demonstrates no significant association with HCC incidence [289].

### Implications for HCC therapeutic stratification and biomarkers

Diseases characterized by IR are closely intertwined with the underlying conditions that predispose individuals to HCC. MASLD occurs in 47.3–63.7% of individuals with T2DM and in approximately 80% of individuals with obesity [290]. T2DM increases the risk of HCC by 2- to threefold [291], while obesity is associated with an approximately twofold elevated risk [292]. These epidemiological patterns suggest that specific metabolic backgrounds in HCC patients, either independently or in combination, may influence liver-related outcomes. Moreover, in patients with HCV who also have diabetes or hyperlipidemia, treatment with metformin or statins significantly reduces HCC risk, suggesting the importance of tailored metabolic management [293]. Thus, in addition to oncological staging, incorporating IR-related factors and associated metabolic diseases into clinical evaluation will probably contribute to a more holistic stratification of HCC patients, enabling personalized treatment strategies and improving the anticipated efficacy of therapeutic interventions.

**Fig. 6:**
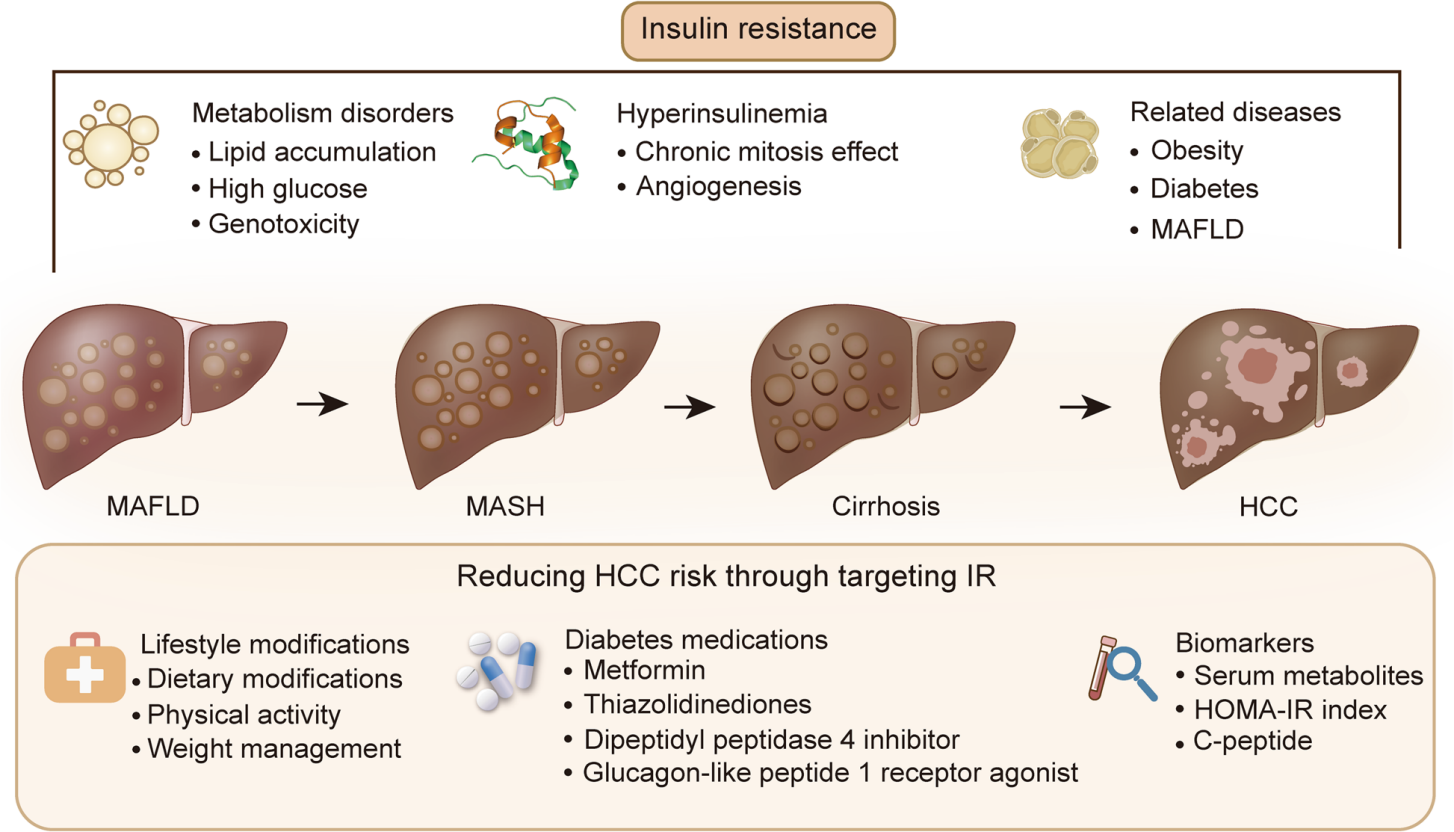
The relationship between insulin resistance (IR) and HCC development, and the potential of reducing HCC risk through targeting IR. MASLD: metabolic dysfunction–associated steatotic liver disease; MASH: metabolic dysfunction-associated steatohepatitis; HCC: hepatocellular carcinoma; HOMA-IR: homeostatic model assessment of insulin resistance

Hyperinsulinemia and metabolic dysregulation resulting from IR offer opportunities to identify novel biomarkers, such as serum metabolites and fasting plasma insulin levels, which can improve HCC risk prediction (Fig. 6). Serum metabolites, including taurine, glycocholic acid, cholesteryl esters, phospholipids, and sphingomyelins, have been shown to exhibit high diagnostic value for HCC versus controls [294]. In a large-scale study, metabolites such as phenylalanyl-tryptophan (Phe-Trp), glycocholate, taurocholate, choline, and taurine are identified as potential biomarkers [295]. Among these, the combination of Phe-Trp and glycocholate has emerged as the optimal biomarker panel for distinguishing HCC patients from those without the disease. Glycocholate is a major bile salt derived from cholic acid and elevated plasma bile acids have been linked to IR, particularly 12α-hydroxylated bile acids, suggesting a potential mechanistic link between IR and bile acid metabolism in HCC pathogenesis [296, 297].

HOMA-IR has been linked to the occurrence of HCC and serves as a strong predictor of liver-transplantation or mortality outcomes [298]. It is also associated with HCC development in patients with chronic HCV infection [299]. Elevated HOMA-IR levels are significantly associated with HCC recurrence, even in patients without MASH/MASLD or diabetes [300]. Triglyceride-glucose (TyG) index can diagnose and predict MASLD, suggesting the importance of monitoring IR in HCC development [301]. C-peptide serves as an indicator of portal insulin secretion, being released in equimolar amounts to insulin and not degraded in the liver, and its elevated circulating levels are related to an increased risk of HCC [302]. In patients with single small HCC, fasting plasma insulin concentration is associated with accelerated HCC growth [303]. Besides, serum lipid parameters, such as high-density lipoprotein cholesterol, are significantly linked to HCC aggressiveness [304]. Additionally, given that systemic inflammation and IR are intertwined, combing the indicators of both inflammation and IR (e.g., C-reactive protein and triglyceride-glucose index) can better predict the prognosis of patients with cancer [305].

## Conclusion and perspectives

Evolutionary conservation of insulin signaling underscores its fundamental role in linking nutrient availability to organismal survival. However, this system is now persistently stimulated in modern life, where overconsumption of high-caloric food and physical inactivity has gradually driven this physiological signaling to an IR pathological state. Chronic inflammation, oxidative stress, elevated toxic lipids and glucose, and subcellular dysfunction, such as impaired mitochondrial activity and ER stress are detrimental to insulin signaling transduction. Notably, metabolic disturbances related to IR, including hyperglycemia, dyslipidemia, and hyperinsulinemia, collectively create a supportive microenvironment that expedites progressive liver lesion. In addition to the metabolic dysregulation, IR-mediated signal transduction can directly or indirectly promote genomic instability, cell proliferation, and tumor angiogenesis, thereby contributing to tumor heterogeneity and malignancy. Since IR is a modifiable condition, its active management through lifestyle modifications and anti-diabetic medications remains essential strategies to improve insulin sensitivity, manage hyperglycemia, and ultimately reduce the burden of chronic liver diseases and their progression. Therefore, understanding the role of IR-factor in HCC stratification is important, as their incorporation may allow better prediction of risk and more precise therapeutic decision compared to relying solely on common phenotypic factors.

Several open questions still remain for investigation. Importantly, for patients with established or advanced HCC, how to create a refined stratification system that identifies specific subgroups most likely to benefit from IR-targeted interventions? For this goal, in addition to the existing staging system, referencing both systemic conditions (e.g., IR, obesity, diabetes) and organ-specific factors (e.g., liver cirrhosis, viral infection) may be more informative when evaluating the etiological impact on treatment decisions. Moreover, developing effective biomarkers reflecting these comorbidities is an urgent need to simplify diagnosis and enable accurate stratification, which requires further prospective trials.

We also acknowledge the limitations of this review, as several relevant areas are not covered in great depth. For example, beyond the role of IR in traditional metabolic tissues, does impaired insulin signaling in other organs contribute to systemic IR and thereby accelerate HCC pathogenesis? Further exploration in these areas will be essential for clarifying the pathophysiological contributions and achieving a more comprehensive understanding of diseases, thereby aiding the development of more effective prevention strategies.

Overall, this review integrates a framework linking interorgan crosstalk interfered IR pathogenesis with systemic metabolic dysregulation, and delineates their pathogenic roles in related morbidities, including HCC initiation. Collectively, this review provides new insights into IR-based therapeutic stratification and intervention strategies, thereby contributing to a holistic approach for reducing the risk of IR-related malignant complications, and potentially supporting more precise patient identification populations and improved clinical outcomes.

## Data Availability

Not applicable.
